# RegulomePA: a database of transcriptional regulatory interactions in *Pseudomonas aeruginosa* PAO1

**DOI:** 10.1093/database/baaa106

**Published:** 2020-12-01

**Authors:** Edgardo Galán-Vásquez, Beatriz Carely Luna-Olivera, Marcelino Ramírez-Ibáñez, Agustino Martínez-Antonio

**Affiliations:** Departamento de Ingeniería de Sistemas Computacionales y Automatización, Instituto de Investigación en Matemáticas Aplicadas y en Sistemas. Universidad Nacional Autónoma de México, Circuito Escolar 3000, Ciudad Universitaria, CP 04510 Ciudad de México, México; Academia de matemáticas, UPN unidad 201, camino a la Zanjita, Nochebuena, CP 71230, Oaxaca de Juárez, Oaxaca, México-visiting researcher at Centro de Altos Estudios de la Mixteca, CALMIX, Oaxaca, México; Academia de matemáticas, UPN unidad 201, camino a la Zanjita, Nochebuena, CP 71230, Oaxaca de Juárez, Oaxaca, México-visiting researcher at Centro de Altos Estudios de la Mixteca, CALMIX, Oaxaca, México; CONACyT-UPN unidad 201, camino a la Zanjita, Nochebuena, CP 71230, Oaxaca de Juárez CP 71230, Oaxaca de Juárez, Oaxaca, México; Genetic Engineering Department, Center for Research and Advanced Studies of the National Polytechnic Institute-Irapuato Unit. Km. 9.6 Libramiento Norte Carretera Irapuato-León, CP 36824, Irapuato Guanajuato, México

## Abstract

We present RegulomePA, a database that contains biological information on regulatory interactions between transcription factors (TFs), sigma factor (SFs) and target genes in *Pseudomonas aeruginosa* PAO1. RegulomePA consists of 4827 regulatory interactions between 2831 nodes, which represent the interactions of TFs and SFs with their target genes, from the total of predicted RegulomePA including 27.27% of the TFs, 54.16% of SFs and 50.8% of the total genes. Each entry in the database corresponds to one node in the network and provides comprehensive details about the gene and its regulatory interactions such as gene description, nucleotide sequence, genome-strand position and links to other databases as well as the type of regulation it exerts or to which it is being subject (repression or activation), the associated experimental evidence and references, and topological information. Additionally, RegulomePA provides a way to recover information on the regulatory circuits of the network to which a gene pertains and also makes available the source codes to analyze the topology of any other regulatory network. The database will be updated yearly, by our team, with the contributions from ourselves and users, since the users are provided with an interactive platform where they can add interactions to the regulatory network feeding it with their respective references.

**Database URL**: www.regulome.pcyt.unam.mx.

## Introduction

*Pseudomonas aeruginosa* PA01 is a metabolically versatile Gram-negative bacterium. It expresses a wide range of virulence factors that allow it to be an opportunistic pathogen of plants and animals (1). As an opportunistic human pathogen, it is capable of causing a wide array of life-threatening acute and chronic infections, particularly in patients with compromised immune defense (2). This bacterium is a major contributor to morbidity and mortality in individuals suffering cystic fibrosis (3, 4). Its ubiquitous occurrence in the environment is due to several factors, including its abilities to colonize multiple environmental niches and to use many environmental compounds as energy sources (5).

The most studied strain of *P. aeruginosa* is called PAO1 and has a genome sequence of 6.2 Mbp with 5570 predicted genes (6). It is characterized by having a predicted repertoire of 550 proteins classified as transcription factors (TFs) and a set of 24 sigma factors (SFs), from which 19 are classified as extra-cytoplasmic factors (ECFs) (7).

The transcriptional activity has a great relevance in this organism because many TFs that respond to environmental conditions allow this bacterium to be an opportunistic infectious agent; the bacterium also shows a high resistance to antibiotics and capability for xenobiotics degradation (8). The repertoire of regulatory interactions in *P. aeruginosa* can be represented in the form of a transcriptional regulatory network, where nodes represent genes and edges represent regulatory interactions (9).

We present RegulomePA, a database that contains biological information on regulatory interactions in *P. aeruginosa* PAO1—this includes the relations between TFs, SFs and target genes.

This manuscript includes the description of the materials and methods followed to collect the data, the computations that were done in order to get the topological information from the network and the description of the architecture of the database and its web interface. Later we show the results of the topological analysis, the discussion of results and finally the future directions.

## Materials and methods

### Data collection and compilation

PubMed and Google Scholar were mined using the following keywords: ‘*Pseudomonas aeruginosa* PAO1’, ‘transcription factor’, ‘transcriptional regulation’, ‘regulatory activation’, ‘regulatory repression’, ‘gene activation’ and ‘gene repression’, specifically from the digitized information between 1989 and 2020. The cumulative hits obtained ∼1800 articles. We visually screened all the abstracts, and ∼800 abstracts were selected for further data mining. The final dataset was obtained from ∼ 200 research articles.

### Search of effective regulatory interactions

We read the selected papers to choose transcriptional regulatory interactions with experimental evidence, the most employed experimental methods reported were DNA foot-printing, gel retardation, protein and DNA mutations, DNA fragment deletion and RNA-seq. The database considers only direct interactions between TFs and target genes in each paper; indirect interactions were not considered in this version.

### Topological analysis

Once having the whole regulatory network, it was considered as a directed graph, where genes are identified as the nodes and the regulatory interactions are described by arrows, with a source and a target gene. We consider that every node *v* has an input *K_in_(v)* degree and an output *K_out_(v)* degree, depending on the number of nodes from which *v* is target or source, respectively. The input degree distribution was calculated by evaluating the relative frequency of appearance for every input degree, without taking into account those with 0 input degree. Cumulative output degree distribution was calculated adding up the cumulative relative frequency of every output degree. Degree *K(v)* was calculated as the sum of input and output degrees for every node. Biological networks present a degree distribution approximate to a power law $P\left( K \right){\ } = {\ }A{K^{ - \gamma }}$, where *A* is a constant that warrants that the *P(K)* values are <1, and the degree exponent $ \gamma $ is usually between 2 and 3 (10). Degree can be considered as the first and intrinsic natural measure of the importance of a node in a network. To deal computationally with a network, some programs like Octave (11) use the adjacency matrix *B*, which is constructed with inputs ${b_{uv}}$ taking ${b_{uv}} = 1$ if there is an arrow with target $v$ and source $v$ and 0 in other cases.

Another important concept in graph theory is the notion of directed path, that is, the sequence of nodes and arrows on the network, such that the initial node of the sequence is not the same as the end node, and no arrows or nodes are allowed to be repeated. On the other hand, a directed cycle is a directed path where the initial and final nodes are the same; the length of the cycle is given by the number of arrows in it. An undirected graph is connected if for any two nodes there exists an undirected path from one node to the other, a network can be composed by several connected components bearing there are groups of genes that interact separately in the network—a measure that could be relevant in the search of biological modules. On the other hand, small modules known as motifs (12) were found; we look for those subnetworks with three and four nodes recognized as recurring regulation patterns in the literature.

We will also deal with centrality measures and other invariants, which are preeminent to identify the most influential nodes in a network. As we already mentioned, the most elementary is the degree centrality (DC), which gives for every node $v$ a measure of the relative connectivity of a node in the network (13); it is calculated as the degree of the node over $n - 1$, this is the maximum possible degree in a network with $n$ nodes. Other centralities addressed in this study are eigenvector centrality, Katz centrality, PageRank centrality, closeness centrality and betweenness centrality.

Eigenvector centrality assigns the importance of a node proportionally to the importance of their neighbors, then a node is important because it is connected to many nodes or because it is connected to nodes with large centralities. The value of this centrality in each node $v$ is obtained by using the adjacency matrix *B*, the largest eigenvalue ${\lambda _n}$ of *B*, and an initial vector ${x_v}\left( 0 \right)$. We get the value in $v$ by the iteration of function ${x_v}\left( {t + 1} \right) = {1 \over {{\lambda _n}}}\mathop \sum \limits_u^{} B{x_v}\left( t \right)$ (14). The sum is over all the nodes $u$ in the network. A connected network ensures that we can obtain a fixed value ${x_v}$ after a finite number of iterations. A usual $x(0)$ in computational algorithms is the eigenvector associated to ${\lambda _n}$.

PageRank centrality is calculated similarly, with the difference that in the previous sum ${1 \over {{\lambda _n}}}$ and $B$ are substituted by a weighted matrix $C$, where the value of the input ${C_{uv}}$ is given by ${{{B_{uv}}} \over {{K_{out}}\left( u \right)}}$ (15).

Katz centrality works for directed networks taking into account the total number of walks between a pair of nodes; it is calculated similarly to eigenvector centrality and pageRank centrality since the total number of walks of length $k$ between two nodes appears in the adjacency matrix ${B^k}$, that is to say, under the iteration of *B*. In this case, ${1 \over {{\lambda _n}}}$ is substituted by a constant $\alpha $, $ {x_v}\left( {t + 1} \right) = \alpha \mathop \sum \limits_u^{} {B_{uv}}{x_u}\left( t \right) + \beta $ (16).

Under the iteration of ${x_v}$, $\alpha $ is converted in ${\alpha ^k}$, which allows the value to be attenuated for the weight as the interaction between two vertices occurs along longer paths, that is to say, ${\alpha ^k} \lt \lt \alpha $.

A different measure of centrality is provided by the closeness centrality and betweenness centrality, which consider the shortest path distance from a node to other nodes.

We use the closeness centrality of node $v$ defined as the reciprocal of the sum of the length of the shortest paths between the node $v$ and all other nodes $u$ in the graph; it is calculated as ${C_{clo}}\left( v \right) = {{n - 1} \over {\mathop \sum \nolimits_{v = 1}^{n - 1} d\left( {u,v} \right)}}$, where $d\left( {u,v} \right)$ is the shortest path distance between $v$ and $u$, and $n$ is the number of nodes in the network.

The betweenness centrality of a node $v$ is the sum of the fraction of all-pairs shortest paths that pass through $v$, it is calculated as ${C_{Bet}}\left( v \right) = \mathop \sum \limits_{s,t \in V}^{} {{\sigma \left( {s,t \vee v} \right)} \over {\sigma \left( {s,t} \right)}}$, where $ \sigma \left( {s,t \vee v} \right)$ denote the number of shortest paths between $s$ and $t$ that use $v$ as an interior node, and $\sigma \left( {s,t} \right)$ is the total number of shortest paths between $s$ and $t$.

Other types of interesting nodes are global regulators, these were found for *Escherichia coli* network by Martínez-Antonio, A. and Collado-Vides, J. (17), after that, by Galán-Vásquez, E., Luna, B. and Martínez-Antonio, A. (9), a *G* coefficient was defined, which indicates if a regulator is more or less global, it is calculated as, $G = {1 \over 4}\left( {{{TFR} \over {{N_{TF}} + {N_{SF}} - 1}} + {{GR} \over {{N_G}}} + {{SF} \over {{N_{SF}}}} + {{CR} \over {{N_{TF}} - 1}}} \right)$ where N_TF_ indicates the total number of TFs (in the known network in each case), N_G_ is the number of non-regulatory genes, and N_SF_ is the number of SFs in the whole network. Additionally, *TFR* denotes the number of TFs regulated by a each TF and, *GR* the number of non-regulatory genes regulated by a each TF; *SF* represents the distinct SFs used by the promoters of genes regulated by each TF; and *CR* represents the number of TFs each TF co-regulates with. In this study we calculate computationally the *G* coefficient and show the most global regulators in the network.

The clustering coefficient is defined by the probability that the neighbors of a node are also neighbors between them, to get this coefficient it was considered the undirected network. For every node $v$ the clustering coefficient of the node is given by ${C_v} = {{2{E_v}} \over {({K(v)})({K(v) - 1})}}$, where ${E_v}$ is the number of edges between the neighbors of $v$ (13).

Finally, we consider the input and output matching index, these are calculated considering that two nodes can be regulated by the same genes or partially the same genes, similarly can regulate the same genes or a proportion of them, though these two nodes are not bound between them. The matching index between two nodes *i* and *j* assesses the sameness between two vertices *u* and *v* based on the number of mutual shared neighbors, it is calculated as ${M_{u,v}} = {{common\,\,neighbors\,\,of\,\,u\,\,and\,\,v} \over {total\,\,number\,\,of\,\,neighbors}}$ (13).

All algorithms were implemented in Networkx from Python (18) and Octave. The following routines are already included in Networkx: degree, clustering coefficient, Eigenvector centrality, connected components, cycles, Katz centrality, pageRank centrality, closeness centrality and betweenness centrality. On the other hand, the following routines were implemented in Octave: G coefficient, motifs, matching index and directed paths.

### Database architecture and web interface

**Figure 1.**
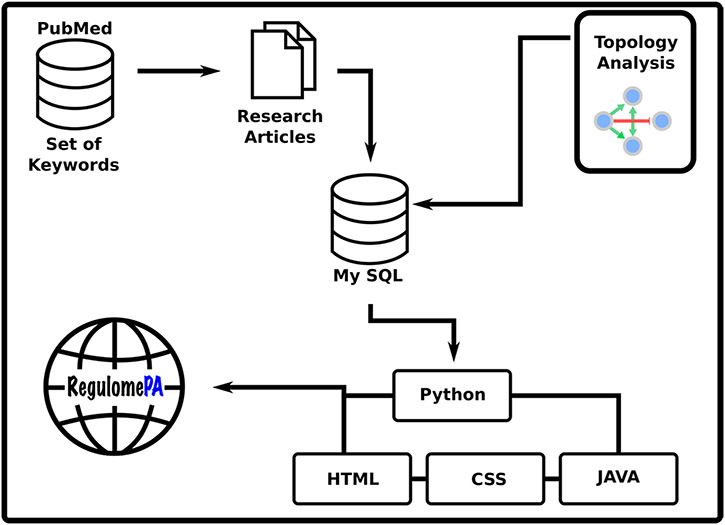
Schema of the RegulomePA database.

RegulomePA was built in Apache server installed in a machine with Ubuntu. MySQL was used as the back end to manage the data, while HTML5, CSS and JAVA scripts were used for developing responsive front ends, compatible for mobiles, tables and desktops. Python 3 using Flask was used as the framework programming language to develop a common interface (Figure 1).

## Results

The regulatory interactions were manually obtained from articles published until 15 September 2020. The regulatory network consists of 4827 regulatory interactions among 2831 gene products, including 163 regulatory proteins and 2668 target genes. Of the 163 regulatory proteins, 150 encode for TFs and 13 for SFs which include 8 ECFs. Considering the 5570 predicted protein coding genes of *P. aeruginosa* PAO1, the current network includes roughly 50.8% of the total genes. 550 proteins classified as TFs covers 27.27% of the expected, and 54.16% of 24 SFs predicted.

### Topology

To characterize the structure of the transcriptional regulatory network of *P. aeruginosa*, we implement several graph theory metrics. In Table 1, we show the general structure of the network, and we compare it with a previous, and first, version of network (9). The interactions from sigma to any other gene, including TFs, were considered as activations, it is worth mentioning that a big contribution of regulatory interactions is given by the house-keeping SF RpoD (19), whose biological activity is transcription initiation. We conserved 13 unknown interactions between SFs, because it is known that in this bacterium, SFs can also act as anti-sigma, blocking the transcription (20). It is notorious that self-regulations are present in around 1.66% of the nodes on the network, which correspond to 28.83% of TF or SF and these are mostly positives.

**Table 1.** General information about the transcriptional regulatory network of *P. aeruginosa*

	Previous network	Current network
Number of nodes	690	2831
Auto-regulations	29 (38.15%)	47 (29.56%)
Positive auto-regulations	16 (55.17%)	24 (51.06%)
Negative auto-regulations	13 (44.82%)	13 (27.65%)
Unknown auto-regulations	-	10 (21.27%)
Regulatory arrows	1020	4827
Positive arrows	779 (76.37%)	3700 (76.65%)
Negative arrows	218 (21.35%)	316 (6.54%)
Dual arrows	11 (1.07%)	8 (0.16%)
Unknown arrows	12 (1.17%)	801 (16.59%)
Maximum out degree	95 (*lasR*)	749 (*rpoD*)
Maximum in degree	8 (*rhlI*)	15 (*rhlR and pvdS*)

**Figure 2.**
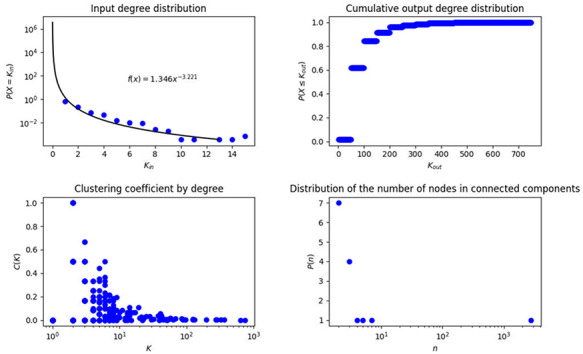
Topological measurements of transcriptional regulatory network of *P. aeruginosa.* (A) Input degree distribution, (B) Cumulative output degree distribution, (C) Clustering coefficient by degree in the network and (D) Distribution of the number of connected components.

In biological networks, degree and degree distribution are two metrics that have gained relevance because they characterize the global structure of the network. In this context, we identified that 45 of the 2831 nodes on the network do not have any input, then, they are just start outputs, 1705 have input degree 1, after that, the frequency of nodes with increasing input degree decreases exponentially until the maximum input degree, which is 15, therefore we cannot adjust the complete behavior to the traditional curves known in the literature, unless we work from input degree 1, then the adjustment is given by the equation $f\left( x \right) = 1.5915{x^{ - 3.117}}$ (Figure 2A). This network is characterized by a small number of highly connected hub nodes and a high number of feebly connected nodes, note that the probability of obtaining a node with input degree 1 is 0.618, in contrast with 0.224 and 0.073, which are the probabilities of obtaining a node with input degree 2 and 3, respectively.

On the other hand, with respect to output degree, a single node influences the other 749 elements (RpoD), while 2668 do not have outputs, that is to say, their output degree is 0, this structure neither fits any known degree distribution, this might be due to the big contribution of the SF RpoD and the fact that many genes are just being regulated without roles of regulators. We construct the cumulative degree distribution (Figure 2B), that is to say, in every value of *x* axis we show the output degree and in *y* axis we show the number of nodes with at most that output degree, this cumulative distribution fits to $f(x) = 0.22{x^{( - 0.379)}}$ (Figure 2B).

The mean of both input and output degree are 1.7051 with a variance of 1.77 and 526.39, respectively, this explains why input degree distribution can be adjusted to a power law, but output degree distribution cannot. Since degree distribution is the sum of both: input and output distribution, the result is dominated by output degree.

Additionally, we identified 15 connected components, with one giant component containing 2790 genes, which represents 98.5% of the genes of this network, while the rest contain from 2 to 7 genes, each connected component contains at least one regulator TF and only the giant component also contains SFs, the number of nodes for each connected component and its frequency is shown in Figure 2C.

Clustering coefficient considers the subjacent network without directions. We found that the highest clustering coefficient is 1, meaning that we found nodes whose neighbors are connected between them forming complete graphs, that is to say, those in which all nodes connect with each other, this characteristic was found for 101 nodes, 1 node with degree 6, 8 nodes with degree 3, and 92 nodes with degree 2, this fact reinforces the appearance of triangles, which was also studied in the analysis of motifs and cycles. The number of nodes with high clustering coefficient constitutes less than 4% of the network. On the other hand, 2401 nodes have a clustering coefficient equal to 0, which corresponds to almost 84.8% of nodes in the network; this is in part due to the fact that 59.2% of the nodes in the network have input degree 1, though we also found nodes with different degrees and clustering coefficients of 0, which are known in graph theory as stars, these are subgraphs with one central node and leaves. The mean of clustering coefficient for the network is 0.06, with a variance of 0.04, this average indicates that neighbors have between them, in mean, 1/25 of connections they could have. The clustering coefficient characterizes small world networks, when this value is big we obtain this type of structure, which is not present in the network analyzed here. The clustering coefficient by degree is presented in Figure 2D in order to better observe the values of this measure.

**Figure 3.**
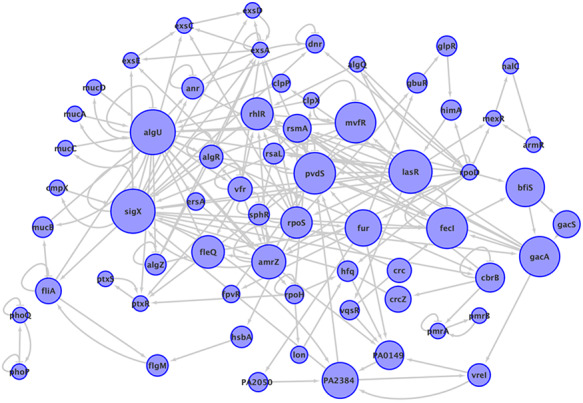
The subnetwork that contains all the elements participating in a cycle in *P. aeruginosa* network, with all the interactions between them. The size of nodes corresponds to the number of cycles to which the node belongs. Two connected components are shown.

The information about paths is included at the database, the regulators used as path starts are 57, among them are ampR, algQ and rpoN. The total number of directed cycles is 65 271, without taking into account the auto regulations, the largest cycle was of size 30, the most abundant cycles were of size 16 with a frequency of 6953 and there are 17 cycles of size 2. The subnetwork composed of all the genes that participate in at least one of the cycles in *P. aeruginosa* network is shown in Figure 3, it contains 63 genes, that is to say, only 2.2% of genes in the network are part of a cycle. The most common genes participating in a cycle are: *algU* present in 63 024 cycles, it follows *sigX* and *lasR*, with 61 515 and 59 200 participations respectively. It is notorious that *rpoD* is present in just one cycle even though its degree is 749, the highest in the network, and it is only feedback by *algQ*. The frequency of cycles by size is shown in the additional material. The subnetwork of cycles in *P. aeruginosa* network is composed of two connected components, one is a cycle of length 2 and the rest of the cycles are fused in the giant component.

**Figure 4.**
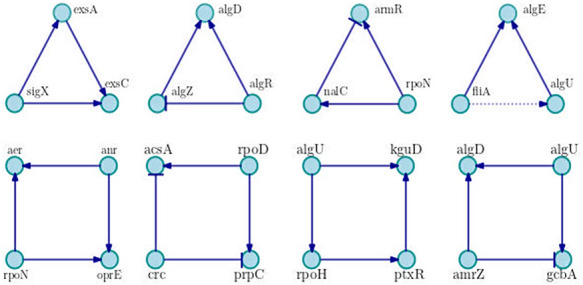
Examples of main motifs of three and four nodes. We show the most frequent motifs in the network, in each case a particular example is shown. Positive interactions are represented by arrows with triangle end, negative interactions are drawn with bar end and unknown interactions are presented in dotted lines.

Relating to motifs of three and four nodes, we compute 679 and 30 626 motifs, respectively. The most recurrent are shown in Figure 4. As can be seen, the network is dominated by positive motifs as the coherent feed-forward loop of three nodes with positive interactions, and the motif of four nodes known as bi-fan, where two TFs each positively co-regulate to two target genes.

The global regulators with the 10 highest values, and their characteristics are shown in Table 2. Compared with those previously reported in 2011 (9), we note that the value of the G coefficient is almost duplicated. Moreover, four of the previous elements remain present in the top 10 though not in the same order, which means certain elements on the network maintain a certain robustness when increasing its size.

**Table 2.** Highest values of G coefficient for TFs

TF	TFs and SFs regulated (excluding self-regulation) TFR	Total of genes regulated GR	Type of sigma used by the regulated promoters SF	Number of TFs used as coregulators CR	G coefficient
*amrZ*	7	107	10	49	0.29535116
*algR*	6	115	9	55	0.28539392
*ampR*	10	18	10	40	0.27654054
*lasR*	10	64	9	40	0.26162011
*gacA*	9	119	8	47	0.25774477
*mexT*	2	48	10	30	0.25022743
*fur*	10	34	9	25	0.233641237
*mvfR*	4	57	8	36	0.225762757
*phoB*	5	35	8	31	0.21685523
*vqsM*	3	58	9	19	0.21502052

Comparing other invariants, we get the results in Table 3, centralities are considered as measures of the importance of nodes, similar to global regulators, though several centrality measures consider an undirected network, we present the top 10 genes with the highest centrality values in each case. As we can see, some genes are consistently repeated in several centrality measures, for instance: *exsA, algU, sigX, fliA, rhlR, pvdS and pqsA.* These regulators are very important intermediates in the network, like canals or great avenues, where regulation fluxes along the network to make the dynamics and physiology of the bacterium possible.

**Table 3.** Highest values of centralities

Degree centrality	Betweenness centrality	Eigenvector centrality	Katz centrality	Closeness centrality	PageRank centrality
*rpoD* (0.265)	*algU* (0.006)	*rhlR* (0.127)	*pvdS* (0.053)	*exsA* (0.011)	*algU* (0.002)
*rpoN* (0.227)	*sigX* (0.005)	*pqsA* (0.127)	*rhlR* (0.052)	*pqsA* (0.011)	*exsA* (0.002)
*algU* (0.130)	*mvfR* (0.004)	*pvdS* (0.122)	*exsA* (0.050)	*rhlR* (0.010)	*pilA* (0.001)
*sigX* (0.116)	*lasR* (0.004)	*exsA* (0.117)	*pqsA* (0.044)	*pqsB* (0.010)	*pvdS* (0.001)
*fliA* (0.100)	*pvdS* (0.003)	*pqsB* (0.18)	*algD* (0.042)	*pqsC* (0.010)	*PA2384* (0.001)
*rpoS* (0.096)	*fliA* (0.002)	*pqsC* (0.108)	*lasR* (0.040)	*pqsD* (0.010)	*gacA* (0.001)
*rpoH* (0.069)	*amrZ* (0.002)	*pqsD* (0.108)	*amrZ* (0.039)	*pqsE* (0.010)	*exsD* (0.001)
*gacA* (0.047)	*rpoS* (0.002)	*pqsE* (0.108)	*rhlA* (0.039)	*exsB* (0.010)	*foxA* (0.001)
*algR* (0.044)	*rhlR* (0.001)	*mvfR* (0.100)	*pqsB* (0.038)	*pvdS* (0.010)	*mexA* (0.001)
*amrZ* (0.044)	*gacA* (0.001)	*rhlA* (0.095)	*pqsC* (0.038)	*exoT* (0.010)	*PA1300* (0.001)

We calculate the input and output matching index, we find 424 626 pairs of genes with input matching index equal to 1, between 8 011 730 possible pairs, this is 5.3%. Other input matching indexes appear for pairs of nodes, for instance 0.5, 0.33, 0.25, 0.16, also 0 appears in the 82% of the possible pairs, when they do not yield any input neighbor. On the other hand, matching index output allows us to detect common TF regulations over the same operons, we present this result with reserve of verification by scientific community, it is necessary to corroborate if it is not the same regulator, in that case it implies robustness of the network, because if one of the regulations fails, the genes still remain regulated by another TF. Only 46 pairs of genes have an output matching index equal to 1, this is 0.00057% of the possible pairs. In fact, there are only seven small groups of genes with exactly the same output neighbors: *amgR, cpxR; clpP, clpX, mucA, mucB, mucC, mucD; deaD, cyaB, exsD; mdrR1, mdrR2; roxR, roxS; PA0149, PA2050* and the group *himA, himD,* which are subunits of the integration host factor nucleoid associated protein. 99.9% of the pairs do not yield any output neighbor.

### Web interface

**Figure 5.**
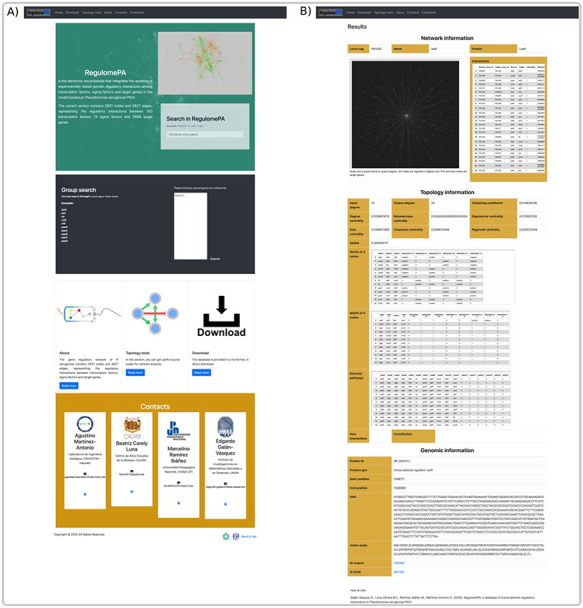
Homepage and gene search at the user interface of RegulomePA. (A) The homepage in RegulomePA, (B) Search results page for a specific gene. As an example, we show the LasR search return: the subgraph of its neighbors, interaction list, topological information, and genomic information.

In RegulomePA the search interface allows two types of queries: the first one is by searching a gene individually either defined by locus tag, gene symbol or protein name; the second is by typing a set of genes (Figure 5A).

The first type of query allows us to obtain the specific information of a gene. It shows the first neighbors of the genes in graph and list form, which include its regulated genes, as well as the TFs and SFs that regulate it. In addition, the main topological measures of the gene are presented, including: input degrees, output degrees, measures of centralities, globality, motifs and paths in which it participates. This will allow us to identify the influence of the gene both, at the global level, considering motifs, regulatory pathways or cascades, and at the local level, because we observe their local neighborhood. Finally, it shows locus tag, gene symbol, protein name, protein ID, gene product, gene sequence, protein sequence and links to PubMed and PRODORIC if more information about that gene is required (Figure 5B).

**Figure 6.**
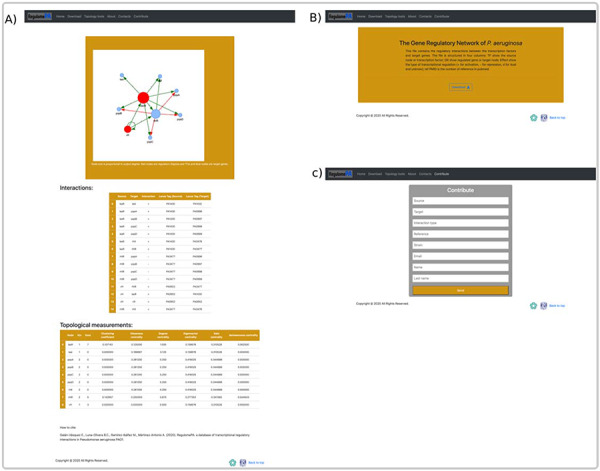
An overview of a search by group of genes, at the user interface of RegulomePA, and other uses of the database. (A) Page of results of the search of a set of genes. As an example, we show the quorum-sensing circuit search return: the subgraph, the list of interactions and the main topological measures of the subnet. (B) Download page for all database and (C) Form to contribute to the database.

The user can explore the regulatory interactions between a set of genes: that may have a similar biological function; to be related to a particular biological process or that was provided from analysis of transcriptomic data. The database returns the set of regulatory interactions in graph and list form. Additionally, it calculates the main topological measures of the subnetworks. This allows identifying regulatory circuits within the network that could serve to identify genes directed toward a construct mutant or overexpressed strains. In the same way, regulatory circuits are used in systems biology to identify attractors by means of Boolean modeling or by differential equations, these reflect biological stages and are related with homeostasis (Figure 6A).

On the other hand, in the download page, you can download the complete list of regulatory interactions in txt format; it is structured in columns that include the input regulator, the target gene, the type of interaction and the reference (Figure 6B). Finally, we provide a template in which the community can contribute by recording the regulatory interactions of *P. aeruginosa* PAO1, in which the regulator, the target gene, the type of regulation, reference, as well as the name and email of the contributor must be typed. These interactions will be validated and added to the network giving credit to the corresponding person or research group. All interactions must be previously published (Figure 6C).

**Figure 7.**
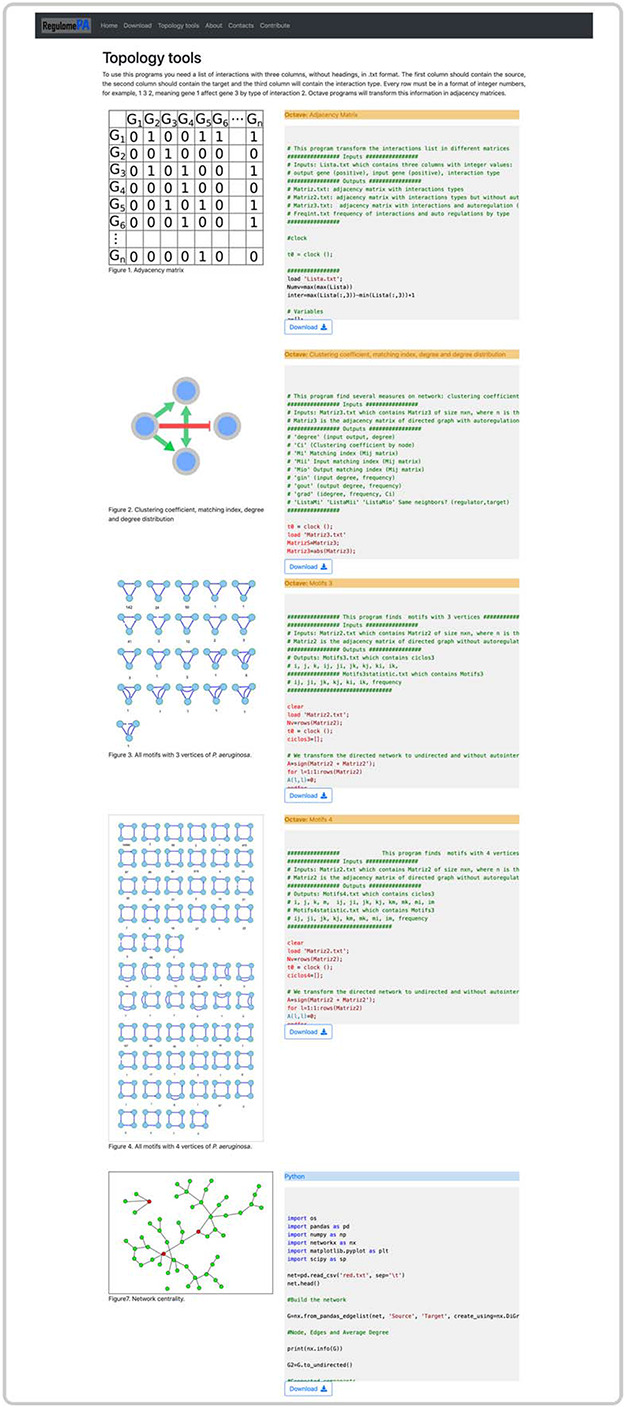
Topological tools at the user interface of RegulomePA. Additional topological characteristics of the network and available programs.

Finally, in the topology page, you can obtain information about the structure of the network which includes degrees of nodes, clustering coefficient, connected components, list of global regulators, list of centralities, cycles, motifs, directed pathways and matching index. In addition, you can obtain the codes for each of the metrics written in Python and Octave (Figure 7).

## Discussion

The transcriptional regulatory network of *P. aeruginosa* has been one of the most important networks in bacteria described in the literature, maybe only after the regulatory network of *E. coli* contained in RegulonDB (21), which is the most complete and the best curated, and the regulatory network of *Bacillus subtilis,* which was reported in 2008 and which has been curated again in 2016 (22).

RegulonDB has been curated for 20 years, this database contains 7127 interactions and 2555 nodes, that includes 211 regulatory proteins, six SFs and 2338 target genes. This network has a cover of ∼69% of regulatory proteins and ∼70% of target genes. In comparison, we have a cover of 27.27% of regulatory proteins, 54.16% of SFs and 50.8% of the total genes, which suggests that we have a good coverage on this first database.

Other approaches in the reconstruction of regulatory networks in particular for *P. aeruginosa* have focused on methodologies based on the gene orthology inference by the reciprocal best hit method as in *P. aeruginosa* strain CCBH4851 (23) and Abasy (Across-bacteria systems) Atlas that integrate regulatory information on different bacteria (24).

Compared with previous studies, RegulomePA has the advantage that it is a database curated from the literature and that every interaction is referenced to specific experiments, in addition to structuring it in a more treatable dataset of the regulatory interactions of *P. aeruginos*a PAO1, which allow us to improve its consultation, downloading and visualization. Additionally, it has a detailed study of the network topology that includes the identification of the most important genes with different metrics, the identification of important subgraphs from a structural and dynamic point of view and the identification of the longest regulatory cascades, also we delve into the treatment of cycles and regulation cascades to obtain more information about the network.

## Future directions

Since RegulomePA database is an interactive platform, one of its updates will come from the academic society interested in this bacterium, and we will present a summary of the updates once a year in the main page. Moreover, we shall work in next directions in the coming years in order to improve the database: curating of high-throughput technologies, integration of expression data, prediction of new interactions with machine learning and DNA-binding site sequences for regulators and for promoters of SFs. Also, expression conditions can be taken into account in order to not only study the network topology but also give insights about the effector signals and rules that govern the dynamical behavior.

## Availability

RegulomePA is freely available at www.regulome.pcyt.unam.mx as a user-friendly, display compatible interface. Additional material like the complete list of motifs on three and four nodes can be found in the same webpage, also the list of sizes of cycles and their frequencies are reported there.
